# Metal Oxide Nanoparticles in Food Packaging and Their Influence on Human Health

**DOI:** 10.3390/foods12091882

**Published:** 2023-05-03

**Authors:** Mariana Stuparu-Cretu, Gheorghe Braniste, Gina-Aurora Necula, Silvius Stanciu, Dimitrie Stoica, Maricica Stoica

**Affiliations:** 1 Faculty of Medicine and Pharmacy, “Dunarea de Jos” University of Galati, 35 Alexandru Ioan Cuza Street, 800010 Galati, Romania; 2 Cross-Border Faculty, “Dunarea de Jos” University of Galati, 111 Domneasca Street, 800201 Galati, Romania; Gheorghe.Braniste@ugal.ro (G.B.); Gina.Necula@ugal.ro (G.-A.N.); 3 Faculty of Food Science, “Dunarea de Jos” University of Galati, 111 Domneasca Street, 800201 Galati, Romania; sstanciu@ugal.ro; 4 Faculty of Economics and Business Administration, “Dunarea de Jos” University of Galati, 59-61 Balcescu Street, 800001 Galati, Romania; stoica_dimitrie2008@yahoo.com

**Keywords:** food packaging, metal oxide nanoparticles, antimicrobials, migration, ingestion, gastrointestinal tract, potential toxicity

## Abstract

It is a matter of common knowledge in the literature that engineered metal oxide nanoparticles have properties that are efficient for the design of innovative food/beverage packages. Although nanopackages have many benefits, there are circumstances when these materials are able to release nanoparticles into the food/beverage matrix. Once dispersed into food, engineered metal oxide nanoparticles travel through the gastrointestinal tract and subsequently enter human cells, where they display various behaviors influencing human health or wellbeing. This review article provides an insight into the antimicrobial mechanisms of metal oxide nanoparticles as essential for their benefits in food/beverage packaging and provides a discussion on the oral route of these nanoparticles from nanopackages to the human body. This contribution also highlights the potential toxicity of metal oxide nanoparticles for human health. The fact that only a small number of studies address the issue of food packaging based on engineered metal oxide nanoparticles should be particularly noted.

## 1. Introduction

**Figure 1:**
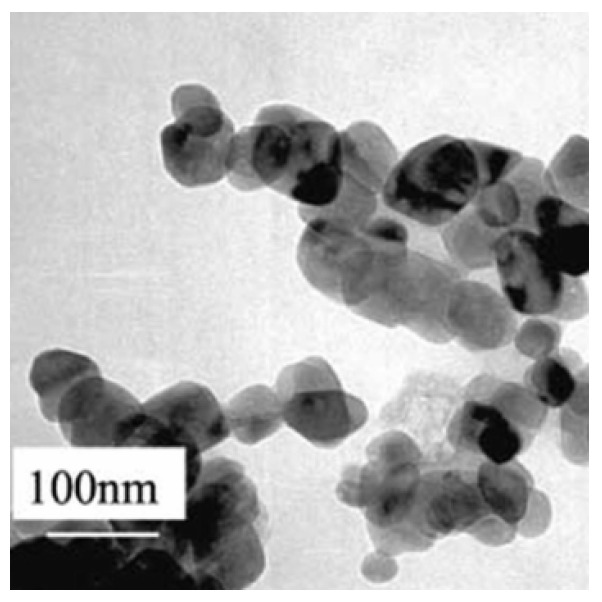
Transmission electron microscopy images of TiO_2_-NPs [25].

We are living in the nanobiotechnology era [1,2]. This newly emerging technology has an enormous potential in various multidimensional fields of modern day life, such as health sciences and engineering (medicine, pharma, sport, agri-food, and other industries) [2,3,4,5,6,7,8,9]. Nanomedicine refers particularly to biosensors, imaging, diagnosis, or drug delivery for therapy, while in sport, nanomaterials (a new class of engineering materials) offer smarter products, such as functional nanosportswear, self-cleaning and antibacterial sportswear and shoes, gadgets, therapeutic bands, rackets, etc. [2,8,10,11]. The agricultural field mainly uses nanomaterials as antimicrobials, larvicidals, insecticidals, nanofungicides and nanofertilizers for their superior efficacy over chemical fertilizers and pesticides [4,12,13]. In the food industry, numerous NPs (nanoparticles) are extensively incorporated into the host polymer/biopolymer matrix to provide strong food packaging characteristics (impactful antimicrobials, biosensors, gas barrier and mechanical strength enhancers, oxygen and water vapor scavengers, etc.) or are directly added into the food matrix to create new food functionalities (coloring, flavoring, safety/stability agents, etc.) [1,4,6,9,14,15,16,17,18,19,20]. Various NPs are used, including nanocellulose, nanoclays, Ag-NPs (nanosilver), Au-NPs (nanogold), nanoforms of some metal oxides (CuO-NPs: copper oxide NPs; Fe_3_O_4_-NPs: triiron tetraoxide NPs; MgO-NPs: magnesium oxide NPs; TiO_2_-NPs: titanium dioxide NPs; ZnO-NPs: zinc oxide NPs), food-grade biopolymers, nanoliposomes, etc. These nanoparticles display unique properties, such as surface effects (larger surface areas, larger surface-to-volume ratios) and pronounced quantum effects due to their nanoscale dimensions (range 1–100 nm), which are directly reflected in their reactivity [1,3,4,6,7,9,15,18,21,22]. The global market size of metal and metal oxide NPs is expected to grow from USD 2.65 billion in 2022 to USD 6.39 billion by 2030, owing to their growing use in various industries (medical sector, pharma, agricultural, food area, and other end-use industries) and increasing awareness of the benefits of using these NPs in different applications [23,24]. Nanoforms of some metal oxides, such as CuO-NPs, Fe_3_O_4_-NPs, MgO-NPs, TiO_2_-NPs, and ZnO-NPs, are used in food nanopackaging owing to their advantages over metal-based nanoparticles (e.g., NPs possess a crystalline structure with more edges and corners [25,26,27,28,29], as can be seen in Figure 1) and their benefits, highlighted in Table 1.

EMo-NPs are effective in enhancing food packaging properties and are excellent antimicrobials owing to their capacity to generate high levels of intracellular ROS (reactive oxygen species) through metal ion release [9,49]. EMo-NPs do not migrate from food/beverage packaging into the food matrix as they are completely encapsulated by the host polymer/biopolymer [16,45,50]. However, in some circumstances, nanostructures (polymer/biopolymer-based matrix) are able to release EMo-NPs into food/beverages, depending on certain factors (food storage conditions, food chemical nature, food processing, etc.) [9,16,45,50]. Once released into the food/beverage matrix, EMo-NPs pass through the human GI (gastrointestinal) tract, cross the GI mucosa and enter human cells, where they display various behaviors. EMo-NPs can be significantly bioaccumulated and exert potential toxicity on human health [1,9,15]. This article offers an insight into the antimicrobial mechanisms of engineered metal oxide nanoparticles as a key benefit in the food/beverage packaging and offers a discussion on the digestive pathway of nanoparticles from contact with food packaging material to the human body. This review also highlights potential toxicity of metal oxide nanoparticles to human health. The article is based on the synthesis of scientific data from the literature, creativity, and knowledge in the field of engineering and health sciences, and it may contribute to understanding the potential toxicity of metal oxide nanoparticles, inspiring researchers to be more concerned with the study of nanopackaging in the food industry.

## 2. Antimicrobial Mechanisms of EMo-NPs

**Figure 2:**
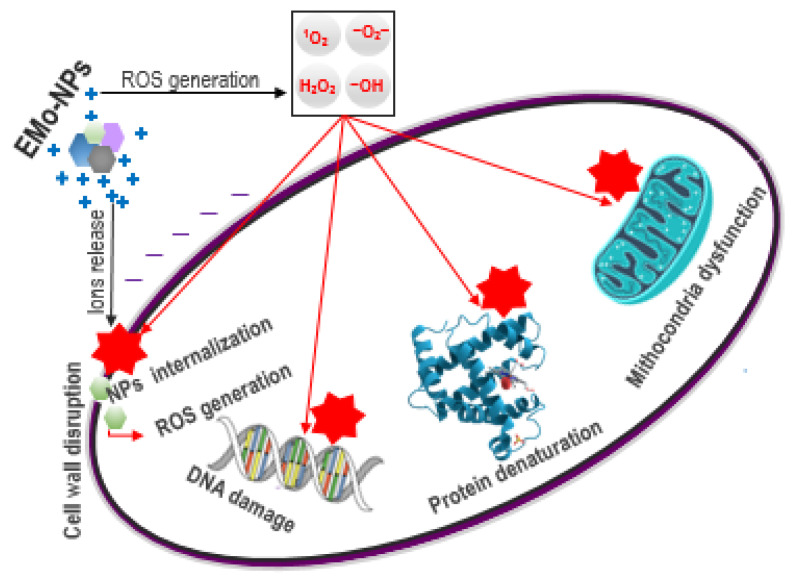
EMo-NPs antibacterial effects: extracellular/intracellular ROS induction (through metal ion release) with subsequent damage of the cell wall, DNA, proteins, and mitochondria. Sources of the cell organelles: “https://knowgenetics.org/dna-mutations-2/, https://www.cleanpng.com/png-biochemistry-biology-protein-science-pathway-1197074/ (accessed on 15 March 2023)”, “https://www.shutterstock.com/ro/search/mitochondrial (accessed on 15 March 2023)”.

EMo-NPs have been used in the food packaging industry for several years due to their benefits, particularly their excellent antimicrobial activity (as shown in Introduction, Table 1) due to ROS induction (extracellular/intracellular) through metal ion release. ROS consists of very cytotoxic radicals O_2_^−^ (superoxide anion radical), ^1^O_2_ (singlet oxygen), OH (hydroxyl radical), and nonradical H_2_O_2_ (hydrogen peroxide) which attack the bacterial cell components (cell wall, DNA (deoxyribonucleic acid), proteins, mitochondria, etc.) [51] (Figure 2).

EMo-NPs can attack numerous microorganisms through ROS induction via Fenton-type and Haber–Weiss reactions, as well as via photocatalytical reactions and NP surface defects [52].

### 2.1. ROS Induction through Fenton-Type and Haber–Weiss Reactions

EMo-NPs release metal ion species which can start Fenton, Fenton-like, and Haber–Weiss Reactions. The Haber–Weiss and Fenton-like reactions can together generate an avalanche of ROS [9,52]. For instance, Cu-based NPs can attack numerous bacteria and yeasts (e.g., *Bacillus*, *Escherichia*, *Pseudomonas*, *Salmonella*, *Staphylococcus*, *Streptococcus*, *Saccharomyces*) due to ROS induction based on Cu^+^ ions’ release [53]. Cu^+^-based ROS induction takes place via Fenton-type (oxidized Cu^+^ interacts with H_2_O_2_, producing ·OH) and Haber–Weiss (induce oxidative stress by generating ·O_2_^−^ and ·OH) mechanisms (Equations (1)–(3)) [54,55,56,57].

(1) $${\mathrm{Cu}}^{2+}+\cdot O_{2}{}^{-}\to {\;\mathrm{Cu}}^{+}+{\;O}_{2}\;\text{(Fenton-like Reaction)}$$

(2) $${\mathrm{Cu}}^{+}+H_{2\;}O_{2\;}\;\to {\;\mathrm{Cu}}^{2+}+\cdot \mathrm{OH}+{\;\mathrm{OH}}^{-}\;\text{(Fenton Reaction)}$$

(3) $$\cdot O_{2}{}^{-}+H_{2\;}O_{2\;}\to \;\cdot \mathrm{OH}+{\;\mathrm{OH}}^{-}\text{(Haber—Weiss Reaction)}$$

(4) Bacteria+·OH →Bacteria damage

Fe_3_O_4_-NPs are also known to catalyze Haber–Weiss reactions [52]. These NPs are a special type of EMo-NP with superparamagnetic and redox properties, possessing very remarkable biocidal activity against various antibiotic-resistant microorganisms (e.g., *Acinetobacter*, *Achromobacter*, *Klebsiella*, *Pseudomonas*, *Serratia*, *Shigella*, *Yersinia*) due to the ROS induction based on Fe^2+^ ions’ release, which are essential for Fenton-like reactions and Haber–Weiss reactions (Equations (4)–(6)) [31,58,59,60,61,62].

(5) $${\mathrm{Fe}}^{2+}+H_{2\;}O_{2\;}\to {\;\mathrm{Fe}}^{3+}+{\;\mathrm{OH}}^{-}\;+\;\cdot \mathrm{OH}\;\text{(Fenton Reaction)}$$

(6) $${\mathrm{Fe}}^{3+}+H_{2\;}O_{2\;}\to {\;\mathrm{FeOOH}}^{2+}+{\;H}^{+}\text{(Fenton-like Reaction)}$$

(7) $$\cdot O_{2}{}^{-}+H_{2\;}O_{2\;}\to \;\cdot \mathrm{OH}+{\;\mathrm{OH}}^{-}\text{(Haber—Weiss Reaction)}$$

(8) Bacteria+·OH →Bacteria damage

Other EMo-NPs, such as MgO-NPs, also have remarkable antimicrobial potential against important foodborne pathogens (*Escherichia*, *Klebsiella*, *Pseudomonas*, *Salmonella*, *Staphylococcus*) by induction of superoxide radicals [63]. While the biocidal mechanism of these NPs is still not clear, it may be due to the following: the generation of more active superoxide radicals outside the microbial cells under light exposure; Mg^2+^ delivery; strong electrostatic interaction with bacterial cells (stronger in Gram-negative strains), causing the disfunctions of cell walls (which could be the crucial mechanism of bacterial destruction); pH change and alkaline effect [51,64,65,66,67,68,69,70]. To sum up, the Fenton and Fenton-like reactions oxidize the metal ions, while the Haber–Weiss reactions reduce the metal ions, inducing bacterial damage [52].

### 2.2. ROS Induction by EMo-NPs through Photocatalytical Reactions

TiO_2_-NPs and ZnO-NPs promote photochemical reactions as they are activated under illumination (visible light/UV light, TiO_2_ being more active in UV light) and many highly reactive pairs ((e^−^/h^+^) (e^−^: electrons, which can act as reductants; h^+^: holes, which can act as oxidants)) are produced (Equation (7)) [9,33,51,52,71,72,73,74]:

(9) $$\mathrm{Metal}\;\mathrm{oxide}\;\overset{\mathrm{hv}}{\to }\;\mathrm{Metal}\;\mathrm{oxide}\left(e_{\left(\mathrm{cb}\right)}^{-}\right)+\left(h_{\left(\mathrm{vb}\right)}^{+}\right)\;$$

where hv is solar light with photonic energy, e^−^_(cb)_ is a conduction band electron, and h^+^_(vb)_ refers to energy for hydrogen bond formation. The (e^−^/h^+^) pairs have a high potential to generate ROS. The mechanism of photocatalytical microbial inactivation for TiO_2_ under solar light are shown in Equations (8)–(11) [75,76]:

(10) $${\mathrm{TiO}}_{2}\left(e_{\left(\mathrm{cb}\right)}^{-}\right)+O_{2\mathrm{ads}}\to \cdot O_{2}{{}^{-}}_{\mathrm{ads}}$$

(11) $${\mathrm{TiO}}_{2}\left(e_{\left(\mathrm{cb}\right)}^{-}\right)+O_{2}+H^{+}\to {\mathrm{HO}}_{2}{}^{\cdot }$$

(12) $${\mathrm{TiO}}_{2}\left(h_{\left(\mathrm{vb}\right)}^{+}\right)+{\mathrm{OH}}^{-}{}_{\mathrm{ads}}\to \cdot \mathrm{OH}\;$$

(13) $${\mathrm{TiO}}_{2}\left(h_{\left(\mathrm{vb}\right)}^{+}\right)+H_{2}O_{2}{}_{\mathrm{ads}}\to \cdot {\mathrm{OH}}_{\mathrm{ads}}$$

TiO_2_-NPs do not deliver metal ions. Their biocidal potential might be attributed to interaction with intracellular biomolecules, adsorbed onto NPs, that possess cytotoxicity, phototoxicity, and ROS induction capacity [53,56,72,77]. In addition, TiO_2_-NPs can induce ROS under dark conditions as a consequence of catalytic reactions from O_2_ and NP surface defects (Equation (12)) [61].

(14) $$O_{2}\;\frac{\mathrm{NP}\;\mathrm{surface}\;\mathrm{defects}}{\to }{\;}^{1}O_{2}\;\;$$

ZnO-NPs are another photocatalytical, possessing high antibacterial action due to their distinctive electronic configuration [53]. ZnO-NPs damage microorganisms by electrostatic forces which damage the cell membrane, Zn^2+^ ions’ delivery, and ROS formation capacity (the most plausible mechanism). ZnO-NPs induce ROS owing to their photocatalytic and UV light photocorrosion (pitting of cell membrane due to ROS) properties [9]. The antimicrobial activity of ZnO-NPs is enhanced by exposure to visible light [1].

## 3. EMo-NPs’ Route from Food/Beverage Packaging into Human GI Tract

### 3.1. EMo-NPs Migration from the Package Matrix into Food/Beverage Matrix: Possible Mechanisms

**Figure 3:**
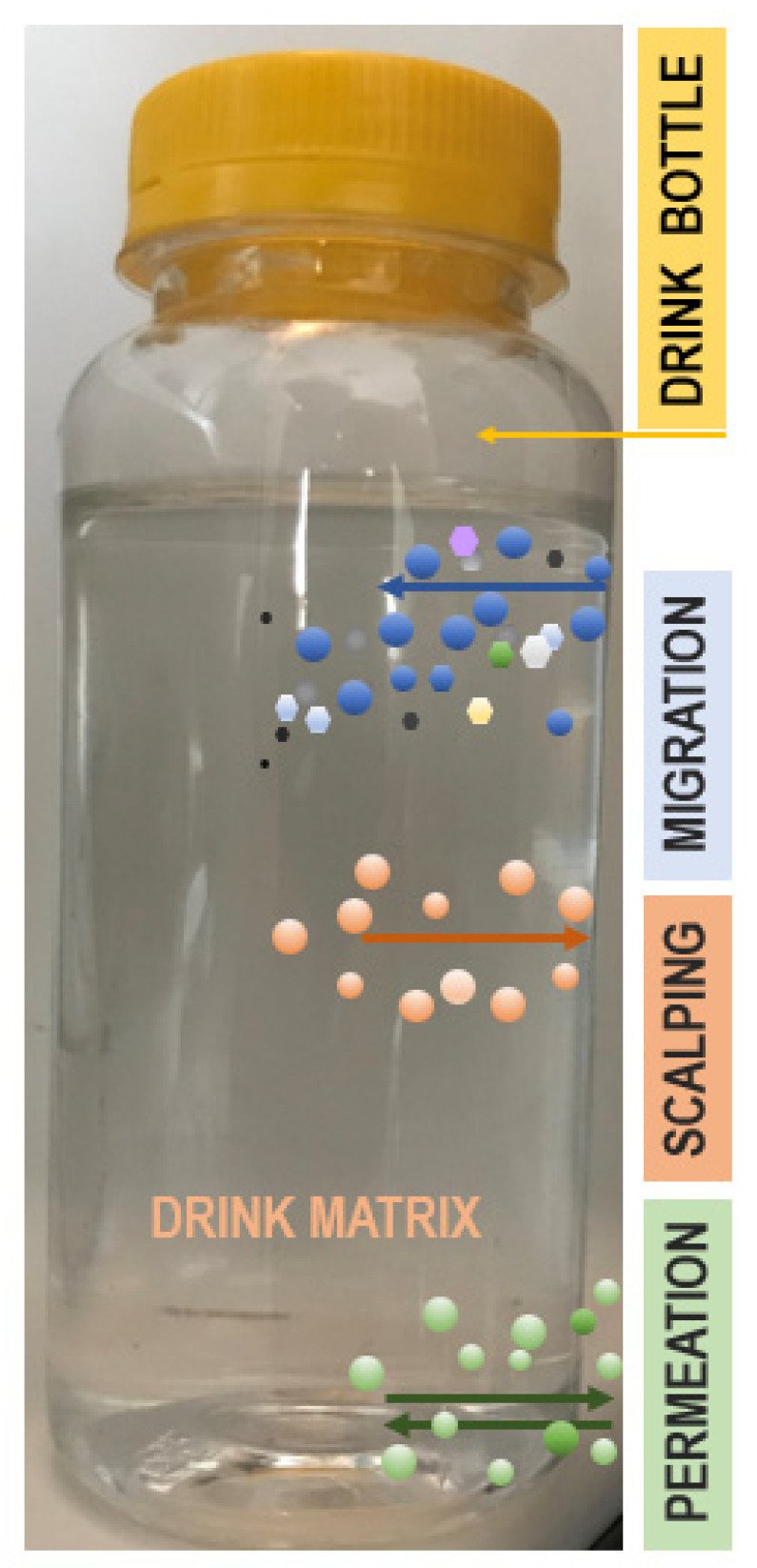
Possible interactions from a polymer-based bottle/drink/external environment. *Migration*: blue circles represent molecular-scale migrants (components from polymer matrix, such as monomers); colored hexagons represent EMo-NPs from polymer matrix. *Scalping*: pink circles represent constituents of drink matrix. *Permeation*: green circles represent gases (CO_2_, O_2_) from both drink matrix and external environment.

This subsection of the article deals with the possible migration mechanisms of EMo-NPs. Innovatively engineered nanopackages, able to contribute to the quality and safety of food/beverages, are viewed as an excellent substitute for conventional packaging composites. Nevertheless, the intentional embedding of engineered nanoparticles, such as EMo-NPs, into the polymer/biopolymer matrix raises some issues, such as the EMo-NPs’ transfer from the package and subsequent release into food/beverages [37,45,78,79,80]. Of all the possible interactions which take place between the nanopackage and the food/beverage contained therein (*permeation*: diffusion of gases across the package wall; *migration*: bulk movement not only of EMo-NPs but also of monomers, antioxidant and coloring agents, printing inks, and antibacterials from the package; *scalping*: uptake of the food/beverage components by the package) (Figure 3), migration is crucial due to the concern that human health might be endangered by the leaching of migrants from the package into the food/beverages [50,81,82,83,84].

EMo-NPs’ release, a highly complex phenomenon, has four potential mechanisms: (desorption: extemporaneous release of NPs bound at the nanopackage interface; diffusion (migration): mass movement (entropically driven) of EMo-NPs from nanopackages and their subsequent release into food/beverage; dissolution: EMo-NPs’ dissolution into ionic species, followed by release of metal ions into food/beverage; EMo-NPs’ releasing as a consequence of any procedure which modifies the host matrix (e.g., photooxidation, abrasion of the host polymer/biopolymer) [6,16,45,50]. EMo-NPs’ (quasi-molecules with evident molecular volume) migration should be ruled by interrelated factors which cause the transfer of conventional molecular chemicals (migrant nature, temperature and contact time, contact type, food chemical nature, etc.), although embarrassing factors may come into play, giving rise to supplemental incertitudes [16,45,85,86,87,88,89]. Nanosize may introduce statistical complexity into migration kinetics; in addition, the rate of EMo-NPs moving through the host matrix is highly impacted when EMo-NPs’ surfaces are coated with surfactants. EMo-NPs take part in complex interactions with the host matrix, and the Fickian diffusion (which obeys Fick’s laws in the case of molecular-scale substances) model cannot be applied. EMo-NPs inserted into the host matrix diffuse from their starting point (dissolved ions, whole) to the interfacial boundary; then, they are released (a notably more complex phenomenon), whereas surface-bound EMo-NPs can be extemporaneously released into the food matrix [45]. Migration depends on properties of EMo-NPs such as the polymer/biopolymer matrix, contact time, contact type, temperature, pressure, and even the manner in which the package is opened. One of the most significant parameters of EMo-NPs is molecular volume [78,90,91]. EMo-NPs that are usually used in materials that come into contact with food are based on polymers larger than 5 nm in size. In addition, they are quasi-immobilized into the host matrix, demonstrating extremely low diffusion even at high levels of NP usage in the polymer/biopolymer. The completely embedded EMo-NPs (covered or encapsulated by the host matrix) do not travel through polymer layers, do not penetrate the outer layer, and do not diffuse into foods [16,45]. However, there is a risk of possible EMo-NP release at or close to the food contact surface in the case of mechanical impact (abrasion) and/or the senescence of the packaging matrix (chemically, mechanically, or thermally stress-induced) [16,45,50]. At a cut edge of food packaging, EMo-NPs will come into direct contact with the food matrix, and the food constituents’ scalping into the polymer/biopolymer package structure occurs more vigorously here. Consequently, EMo-NPs will be dissolved by food constituents or may even be physically released, again with the possibility of being dissolved [16,45,86]. The migration measurements of EMo-NPs or metal ions inside the food matrix are difficult to carry out (EMo-NPs are chemical chameleons, appearing in various size and shapes and sometimes dissolved into ions or reduced back into their constituents; in addition, food/beverage is a very complex matrix) but are essential to determine their possible health implications, as they are often considered to have toxic potential [6,16,53].

### 3.2. EMo-NPs’ Route from the Food/Beverage Matrix into the Human GI Tract

**Figure 4:**
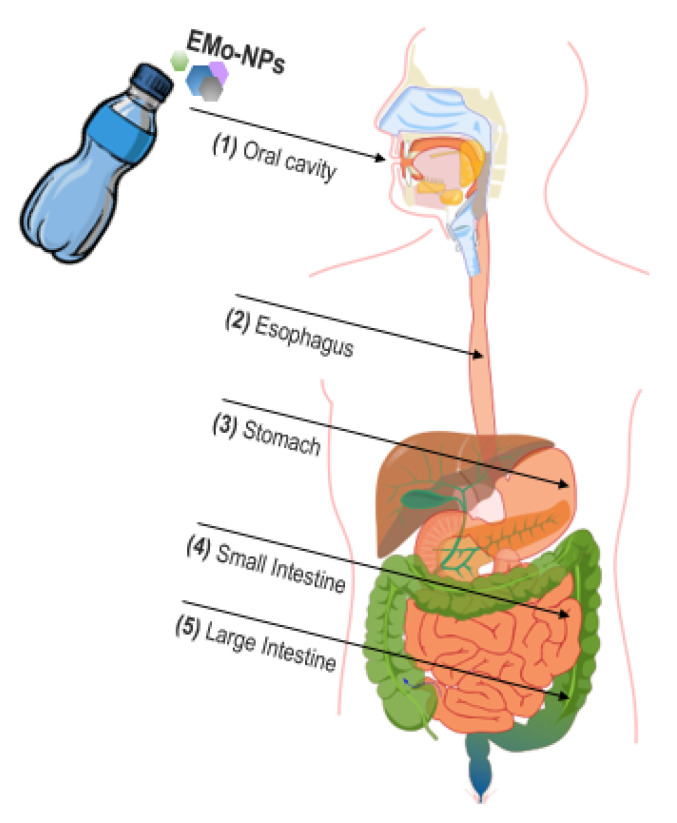
The oral route of EMo-NPS into the human GI tract. Source of the human digestive system: “https://commons.wikimedia.org/wiki/File:Digestive_system_without_labels.svg. (accessed on 20 March 2023)”.

The human body is easily poly-exposed to EMo-NPs via oral (ingestion), dermal, and respiratory (inhalation) routes [4,6,92,93,94]. Food/beverage ingestion is the primary route of EMo-NPs’ absorption [94]. EMo-NPs enter into food/beverage formulation through transfer out of nanopackaging or nanopackaging headspace or as direct food additives (fortifying agents, dietary supplements, coloring agents, etc.) [1,9,15,92,95]. EMo-NP_S_ dispersed into the food/beverage matrix enter the human GI tract after ingestion (Figure 4), where they display various behaviors.

The absorption of EMo-NPs could start in the oral cavity (1) through buccal and sublingual mucosa (vastly permeable), directly passing into the circulatory system and, subsequently, penetrating into cells through fine capillaries [92]. Unabsorbed EMo-NPs pass through the oral cavity (characterized by approximately neutral pH, electrolytes, metabolic enzymes, biopolymers, and mastication), move through the esophagus (2) to the stomach (3) (characterized by highly acidic pH, around 2–3; electrolytes; enzymes and biopolymers; peristalsis; and churning), and enter into the small intestine (4) (pH 5–7; electrolytes; bile salts; enzymes and biopolymers; and peristalsis). Once ingested, EMo-NPs can adhere to, travel through, or be adsorbed by the mucus layer of the GI tract. If EMo-NPs are not absorbed in the (2–4) segments of the GI tract, they are released into large intestine fluids (5) (pH 7–8) as the food matrix is disrupted and digested [15,96]. EMo-NPs adhere to enteric mucosa (the first barrier—a complex hydrogel consisting mainly of mucin proteins, negatively charged, influencing the adhesion of EMo-NPs through electrostatic interactions) and diffuse through it; then, they are absorbed by the chylomicron uptake mechanism of enterocytes and transported across the epithelium (the second barrier with the highest resistance against EMo-NPs’ passage) [92,96]. EMo-NPs are taken up by active (transcellular) or passive (paracellular, with a minor role in the passage of NPs) mechanisms into cells (or even on the subcellular level) and various organs (brain, heart, intestines, kidneys, liver, lungs, spleen, and stomach). The liver and spleen absorb and accumulate EMo-NPs significantly faster than others [15,92,97]. EMo-NPs are not metabolized in the GI tract and can be absorbed in their intact form (being bio-persistent) [1,6,15,94]. The large pH gradient, electrolytes, physical forces, and characteristics of EMo-NPs are significant parameters that may impact EMo-NP interactions with various biomolecules originating from the ingested food/beverage or GI tract. The significant modification of the interfacial properties of EMo-NPs that subsequently occurs could influence biological tissues and cellular response. EMo-NPs have a highly specific surface area, providing a large area for the adsorption of any surface-active components such as bile salts, metabolic enzymes (amylases, lipases, peptidases), proteins, or phospholipids (resulting in a reduction in their activity) [15,96]. EMo-NPs in high concentrations could decrease the digestion of lipids, proteins, and starch within the GI tract, alter the normal function of the epithelial cells of intestinal tissue and nutrients’ absorption, and stimulate an immune response with potential adverse effects on human health [1,15]. The properties of ingested EMo-NPs are also significantly modified by bacteria from the human GI tract through secreted enzymes and biopolymers. Conversely, ingested EMo-NPs demonstrate complex antimicrobial activity that could impact the colonic microbiota (which has an important role in the maintenance of the structural integrity of the mucosal barrier) and alter the host’s physiology, exerting a powerful influence on wellbeing and human health [6,15,98].

## 4. Influences of EMo-NPs on Human Health

### 4.1. Intracellular ROS Induction in Eukaryotic Cells

**Figure 5:**
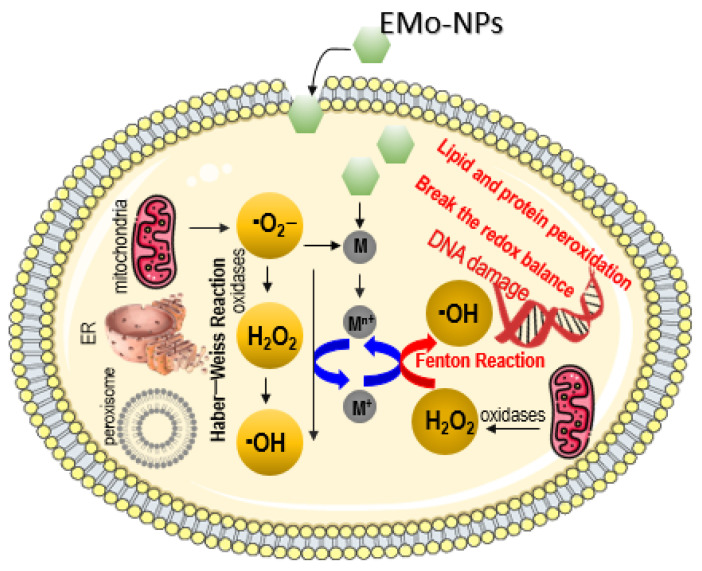
ROS induction inside the eukaryotic cell by EMo-NPs. EMo-NPs are much more toxic than metal ions, serving as *Trojan horse-type carriers* (green hexagons) which release metal ions inside the cell (gray circles). Source of the cell membrane: “https://smart.servier.com/smart_image/cell (accessed on 22 March 2023)”. Sources of the organelles: “https://www.pngwing.com/en/search?q=mitochondria (accessed on 22 March 2023)”, “https://commons.wikimedia.org/wiki/File:201601_Endoplasmic_reticulum.png, (accessed on 22 March 2023)”, “https://pixabay.com/ro/vectors/dna-biologie-pictogram%C4%83-rna-6177695/ (accessed on 22 March 2023)”.

This subsection discusses the potential toxicity effects of EMo-NPs, especially those involved in subcellular ROS generation. ROS are produced by various biological entities such as cell membrane and subcellular compartments (oxidative organelles: ER (endoplasmic reticulum), mitochondria, peroxisome). These mitochondria generate approximately 90% of cytoplasmic ROS (with a favorable role in cell physiology at low or moderate levels). At the same time, the mitochondria maintain a balanced amount of ROS in the cell as soon as they are generated. Excessive ROS production results in oxidative stress (cell damage, cell death) [99,100,101]. EMo-NPs are extremely reactive; they generate excessive levels of intracellular ROS, which can have effects on the overall functionality of the cell, oxidizing/damaging the cellular biological macromolecules (DNA, lipids, proteins, and enzymes) and biological entities, including the cell wall and organelles [6,15,53,94,97,102] (Figure 5).

In the cell, ROS are natural byproducts of normal oxygen metabolism, being generated by mitochondria during physiological (or pathological) conditions [99,103]. The ·O_2_^−^ radical generated inside the cell by mitochondria is changed by oxidases (superoxide dismutase) into H_2_O_2_, which is then changed into very reactive ·OH radical through Haber–Weiss or Fenton-type reactions. The endogenous ROS is kept in balance by numerous intracellular antioxidant mechanisms. Conversely, in the presence of metal ions released from internalized EMo-NPs, H_2_O_2_ can be rapidly converted to high a ·OH level through Fenton reaction [103]. Excessive generation of ROS by external input of EMo-NPs can break the redox balance and may lead to harmful effects, such as DNA damage, lipid and protein peroxidation, cellular apoptosis, mitochondria dysfunctions (via depolarization of mitochondrial membrane), as well as other related phenomena, such as cellular signaling fluctuations involved in cell differentiation and cell proliferation [99,104].

### 4.2. EMo-NPs: Main Concerns

The data on possible EMo-NP toxicity to humans are limited. Most studies related to EMo-NP toxicity were conducted on various in vivo models: animals, aquatic organisms, simulated body fluids (saliva, stomach, and intestinal fluids), and human blood plasma. A summary of the main concerns is shown in Table 2.

### EMo-NPs’ Immunotoxicity

EMo-NPs’ immunotoxicity has been revealed (Table 2). Whether EMo-NPs are recognized by the immune system or not, they influence the human immune system (immune organs, cells, molecules) through different immune reactions such as: oxidative stress (as can be seen in Section 4.1), inflammatory/anti-inflammatory responses, and genotoxicity [154,155,156]. Inflammation, the immediate natural response of the body against external chemicals, is favorable for human health; however, uncontrolled inflammation can lead to severe disorders [155,157]. EMo-NPs can induce the release of cytokines/chemokines in tissues and organs (e.g., spleen, liver), which have an important role in controlling and promoting of inflammatory response [155]. The immune system activates phagocytic cells; for instance, polymorphonuclear neutrophils, which migrate to an inflammatory site and induce inflammatory mediators, recruiting more polymorphonuclear neutrophils and other immune cells such as macrophages and lymphocytes. Macrophage cells exert an initial reaction to EMo-NP exposure. They initiate and propagate an inflammatory response with their capacity to recognize/engulf/digest EMo-NPs. Macrophage cells’ interaction with EMo-NPs usually results in activation of NADPH oxidase, leading to ROS induction along with oxidative burst [158,159]. EMo-NPs can also produce oxidative organelle (ER, mitochondria, lysosome) damage or dysfunction in immune cells owing to direct NP accumulation or indirect subcellular changes [160]. ER, the largest organelle, is responsible not only for protein synthesis and lipid metabolism (mainly) but also for upregulating cell response to stress. EMo-NPs may lead to protein misfolding, which induces ER stress [161]. EMo-NPs can also interact with mitochondria, leading to impaired mitochondrial function (mitochondrial stress) after their internalization, which may induce some metabolic disorders and reduce overall cellular energy levels [162,163,164]. EMo-NPs can typically accumulate in lysosomes (endpoints of the endocytosis pathway which act as digestive organelles and are essential for maintaining cellular homeostasis) and lead to impaired lysosomal dysfunction (lysosome membrane permeabilization, resulting in mitochondrial outer membrane permeabilization that induces ROS generation and apoptosis; massive lysosome membrane permeabilization can produce cytosolic acidification and necrosis) [165,166]. EMo-NPs’ genotoxicity has also been revealed (Figure 5, Table 2). Its genotoxicity is primarily size dependent: the smaller the size, the higher the reactivity of the surface area and, therefore, the higher the ROS induction, genotoxic reactions, and DNA damage [9,167,168]. The degree of severity may be closely related to the oxidative stress caused, and it is also dependent on EMo-NPs’ concentration and their physicochemical features. However, owing to the influence of key factors related to the toxicity of EMo-NPs (size, shape, morphology, surface coating, surface reactivity, specific surface area, solubility, bonded surface species, oxidation status, agglomeration/aggregation degrees), the relevant immunotoxicity mechanism is not completely understood. Long-term systematic studies are required to explore this further and to clearly explain the interaction between EMo-NPs and human tissues [155].

## 5. Conclusions

Engineered metal oxide nanoparticles are dominant and promising in the food sector. They are employed in innovative food packaging as impactful antimicrobials, biosensors, and gas barrier and mechanical strength enhancers, and in the functional food field as coloring, flavoring, and safety/stability agents. This review offers an insight into the antimicrobial mechanism of engineered metal oxide nanoparticles as a key benefit in food/beverage packaging, with a focus on extracellular/intracellular ROS induction as a primary antimicrobial mechanism (via Fenton-type, Haber–Weiss, and photocatalytical reactions). The paper also highlighted the migration of engineered metal oxide nanoparticles from the package matrix into the food/beverage matrix and their route from the food/beverage matrix into the human GI tract. Engineered metal oxide nanoparticles for food packaging are completely embedded in the host matrix. They do not possess the potential to travel through the polymer layers to the food contact layer where transfer into the food could occur. However, in certain circumstances (chemical, mechanical, or thermal stress-based package) a series of interactions (such as migration) may occur between the food product and its package, permitting the nanopackaging to transfer the desorbed nanoparticles into the food product. Once migrated into foods, nanosized metal oxide particles can enter into the GI tract (via the oral route) and, from there, can readily enter human cells through the circulatory system. Then, the nanoparticles can be absorbed by biological tissues, causing adverse influences on human health due to excessive intracellular ROS induction. Excessive ROS induction by external input of engineered metal oxide nanoparticles results in negative effects on the overall functionality of the cells and may lead to DNA damage, lipid and protein peroxidation, cellular apoptosis, mitochondria dysfunctions, and cell proliferation. This article does not conclude that engineered metal oxide nanoparticles should not be used; rather, sufficient attention should be paid to their use for food packaging as they may produce undesirable consequences for human health. Furthermore, innovative tests are necessary to obtain a clear illustration of their migration, behavior, and toxicity, and to obtain a better comprehension of the eventual health hazards related to overexposure to nanoparticles. We hope that this article inspires life science researchers, helping them to identify some aspects related to metal oxide nanoparticles so that their use does not pose any risk to human health.

## Figures and Tables

**Table 1:** Types of EMo-NPs and their benefits in the food industry.

Type	Benefits	References
CuO-NPs	Excellent antibacterial (against Gram-positive and Gram-negative bacteria), antifungal, and antiviral agent; good reinforcing NPs of polymer/biopolymer matrix	[1,3,4,9,17,30,31,32,33,34,35,36,37,38,39,40,41,42,43,44,45,46,47,48]
Fe_3_O_4_-NPs	Distinct type of NPs with superior properties compared to α-Fe_2_O_3_ (ferric oxide–hematite) and γ-Fe_2_O_3_ (ferric oxide–maghemite); possesses strong antibacterial characteristics; source of bioavailable iron; mineral-fortified supplement	
MgO-NPs	Strong antimicrobial action similar to nanosilver (depending on their sizes; when the size is smaller than 15 nm, MgO-NPs have a powerful biocidal efficacy); nanoscale MgO is a polymer/biopolymer reinforcement agent; dietary supplement	
TiO_2_-NPs	Top EMo-NPs (major promising NPs player in the food industry, extensively used); photocatalytic antimicrobial activities against bacteria, yeast, and fungi; UV-protective food nanopackaging; enhancer of mechanical and thermal stability of food packaging; oxygen scavenger; biosensor for volatile organic compounds; food coloring agent	
ZnO-NPs	Excellent photocatalytical and photocorrosion antimicrobial against bacteria, yeast, and fungi; UV light absorber in food packaging; source of zinc in food supplements	

**Table 2:** Main concerns related to EMo-NPS.

EMo-NPs	Main Concerns	References
CuO-NPs	Cu, a trace element, is vitally important, playing a significant role in numerous biological activities (hemoglobin production, iron metabolism, hormone synthesis, etc.). However, a high level of Cu ions could be toxic. Cu ions are redox-active, affecting biological systems. Their reactivity is wholly dependent on extrinsic and intrinsic factors (size, shape, surface charge, concentration of NPs). The smaller the size, the more toxic they are. NPs smaller than 40 nm can directly enter cell nuclei from the circulatory system, while NPs greater than 100 nm can cross the cell membrane. Spherical ones are more reactive. CuO-NPs are *Trojan horse-type carriers*, releasing Cu ions inside the cells. Compared with Fe_3_O_4_-NPs, ZnO-NPs, and TiO_2_-NPs, CuO-NPs are the most potent in terms of cytotoxicity. CuO-NPs’ uptake in many organs (spleen, liver, kidneys, brain, lungs, blood, heart, stomach, bones, marrow), and they could exert: oxidative stress genotoxicity cytotoxicity immunotoxicity inflammation	[104,105,106,107,108,109,110,111,112,113,114]
Fe_3_O_4_-NPs	Fe is an essential biological trace element not only for human beings but for all other life forms. In the human body, the majority of Fe is in hemoglobin (50–60%); 25% is in an easily mobilizable store, and the remaining 15% is in myoglobin and in numerous enzymes involved in oxidative metabolism and many other cellular activities. In addition, Fe_3_O_4_-NPs is used in various biomedical applications (cancer, diabetes, diagnosis of contrast substances, magnetic resonance imaging, inflammatory diseases, targeted drug delivery, gene therapy, biosensors, etc.). Although Fe-based supplements are highly effective for improving iron status and Fe_3_O_4_-NPs-based biomedical applications have good potential, there are controversial results regarding the cytotoxic effects and the overall integrity of the cells, once the engineered Fe_3_O_4_-NPs are inside the cells. Fe_3_O_4_-NPs are less toxic than other metal oxide NPs, but they could effectively enter the cell nucleus. Along similar lines as CuO-NPs, Fe_3_O_4_-NPs reactivity is linked to surface modification, concentration, size, shape, dose dependency, obtainment method, etc., and could induce: disruptions of the oxidative balance cytotoxicity immunotoxicity neurobehavioral toxicity inflammation ferroptosis fibrosis/cirrhosis and loss of liver function	[61,115,116,117,118,119,120,121,122,123,124]
MgO-NPs	Mg^2+^ is an important cation for human health. In the human body, the majority of Mg is mainly stored in bones (50–65%) while 34–39% is in muscle, soft tissues, and organs and 1–2% is in blood and extracellular fluids. It plays a significant function in many physiological processes. MgO-NPs are used in a wide range of biomedical applications (cancer therapy, medical imaging, nanocryosurgery, bone regeneration, biosensor, tissue engineering, dental implants, bioactive glasses, etc.). However, MgO-NPs’ toxicity is controversial, depending on the physical and chemical characteristics of the NPs and tested cell type. Concerns about their safety remain (at high concentrations), and refer to: oxidative stress hemolytic activity arteriosclerosis inflammation neronal apoptosis hepatocytotoxicity	[125,126,127,128,129,130,131]
TiO_2_-NPs	TiO_2_-NPs are one of the most commonly used NPs in consumer products (foods, medicines). The exposure of humans to TiO_2_-NPs via the oral route is inevitable. The potential toxicity of TiO_2_-NPs is addressed by multiple studies and is wholly dependent on their size, shape, surface charge, concentration, and solubility. Due to their smaller size, TiO_2_-NPs are more easily absorbed into cells, where they can become involved in: oxidative stress genotoxicity cytotoxicity immunotoxicity inflammation TiO_2_-NPs promote photochemical reactions, as they are more active in UV light, but they can induce ROS under dark conditions. If ROS induction can occur under dark conditions, it can likely take place inside the human body. This may lead TiO_2_-NPs to be toxicologically potent than previously known.	[52,132,133,134,135,136,137,138,139,140,141,142,143,144,145,146,147,148,149]
ZnO-NPs	Zn is one of the most essential trace minerals and possesses exceptional properties such as the capacity to modulate immune responses, improve fertility and metabolism, scavenge free radicals, etc. ZnO-NPs are one of the most prevalent metal oxide NPs and possess wide biomedical applications (treatment of various kinds of cancers, drug delivery, etc.). The exposure of humans to ZnO-NPs is very frequent and constitutes an issue of concern to health. The potential toxicity of ZnO-NPs is entirely dependent on their size, shape, surface charge, concentration, and solubility. Studies of their toxicology in in vivo models indicate that ZnO-NPs may cause: oxidative stress genotoxicity cytotoxicity inflammation hepatotoxicity	[150,151,152,153]

## Data Availability

No new data were created or analyzed in this study. Data sharing is not applicable to this article.
